# Diagnostic Efficacy of 11 SARS‐CoV‐2 Serological Assays for COVID‐19: A Meta‐Analysis and Adjusted Indirect Comparison of Diagnostic Test Accuracy

**DOI:** 10.1002/iid3.70114

**Published:** 2024-12-19

**Authors:** Ying Zhao, Minjie Zhang, Weiwei Liang, Lijiang Fang

**Affiliations:** ^1^ Department of Medical Laboratory Xian Yang Central Hospital Xianyang China; ^2^ Department of Medical Laboratory The Affiliated Hospital of Shaanxi University of Chinese Medicine Xianyang China; ^3^ Department of Medical Laboratory The Second Affiliated Hospital of Shaanxi University of Chinese Medicine Xianyang China

**Keywords:** adjusted indirect comparison, COVID‐19, meta‐analysis, SARS‐CoV2, serological assays

## Abstract

**Objective:**

In the past 5 years, a large number of serological assays for large‐scale detection of antibodies against severe acute respiratory syndrome coronavirus 2 (SARS‐CoV‐2) antigen emerged. Serological assays for SARS‐CoV‐2 were needed to support clinical diagnosis and epidemiological investigations. However, there were limited data on the diagnostic accuracy of these serological assays. We aimed to compare the diagnostic accuracy of 11 commercial serological assays for coronavirus disease‐2019 (COVID‐19) by taking the reverse transcriptase polymerase chain reaction (RT‐PCR) assays as the reference standard, which served as the control arm to conduct an indirect comparison of diagnostic accuracy for 11 different SARS‐CoV‐2 serological assays.

**Methods:**

This meta‐analysis was conducted following the PRISMA 2020 reporting guideline. Electronic searches were performed using the Cochrane Library, PubMed, Embase, Web of Science, Chinese Biological Medicine Database (CBM), China National Knowledge Infrastructure (CNKI), WANFANG, and Chinese Weipu (VIP) databases. Fifty‐seven articles, including 11 serologic‐based IgG, IgM, and total antibodies assays for SARS‐CoV‐2, published before June 2024, were included in this meta‐analysis. The main outcome of this meta‐analysis used to evaluate the performance of 11 assays included pooled diagnostic odds ratio (DOR), area under the summary receiver operating characteristic (AUC), and summary receiver operating characteristic curve (SROC). The R software was used for adjusted indirect comparison to calculate the relative diagnostic odds ratio (RDOR) with corresponding 95% confidence intervals (CIs), and indirect comparison forest plots showed the results.

**Results:**

A total of 57 articles met the eligibility criteria for inclusion in our meta‐analysis. The pooled DOR and the AUC for access SARS‐CoV‐2 IgG were 564.28 (95% CI 229.58−1386.91) and 1.00, and as for EDI novel coronavirus COVID‐19 IgG those were 85.27 (95% CI 53.99−134.68) and 0.95, for EDI novel coronavirus COVID‐19 IgM were 49.42 (95% CI 16.47−148.30) and 0.86, for iFlash‐SARS‐CoV‐2 IgG were 652.31 (95% CI 362.32−1174.41) and 0.97, for iFlash‐SARS‐CoV‐2 IgM were 36.72 (95% CI 12.42−108.54) and 0.76, for MAGLUMI 2019‐nCoV IgG were 145.44 (95% CI 59.37−356.30) and 0.90, for MAGLUMI 2019‐nCoV IgM were 21.59 (95% CI 14.27−32.67) and 0.59, for ortho‐clinical anti‐SARS‐CoV‐2 IgG were 719.46 (95% CI 262.34−1973.13) and 1.00, for ortho‐clinical anti‐SARS‐CoV‐2 total were 1104.60 (95% CI 395.64−3083.99) and 1.00, for Siemens SARS‐CoV‐2 total (COV2T) were 1143.37 (95% CI 316.49−4130.62) and 0.99, for Wantai SARS‐CoV‐2 total Ab were 1014.98 (95% CI 618.48−1665.66) and 1.00. The pooled DOR for assays‐based IgG (*n* = 43), assays‐based total antibody (*n* = 35), and assays‐based IgM (*n* = 20) was 242.88 (95% CI 157.66−374.16), 1215.90 (95% CI 547.14−2702.07), and 40.99 (95% CI 22.63−74.25). The diagnostic accuracy of assays‐based total antibody performed better than those of assays‐based IgG and assays‐based IgM; assays‐based IgG performed better than assays‐based IgM.

**Conclusion:**

This study suggested that the Siemens SARS‐CoV‐2 total (COV2T), ortho‐clinical anti‐SARS‐CoV‐2 total, and Wantai SARS‐CoV‐2 total had the best overall diagnostic accuracy. The diagnostic efficacy of the assays‐based total antibody had statistically significantly higher accuracy than those of assays‐based IgG and assays‐based IgM for COVID‐19.

## Graphical abstract

We aimed to compare the diagnostic accuracy of 11 commercial serological assays for coronavirus disease‐2019 (COVID‐19) by taking the reverse transcriptase polymerase chain reaction (RT‐PCR) assays as reference standard, which served as control arm to conduct an indirect comparison of diagnostic accuracy for 11 different SARS‐CoV‐2 serological assays.

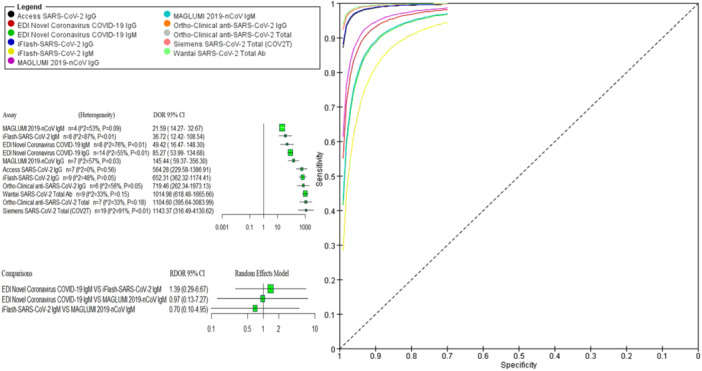

## 1 Introduction

Severe acute respiratory coronavirus 2 (SARS‐CoV‐2), a novel coronavirus that caused coronavirus disease 2019 (COVID‐19), has become a pandemic threat in which serological testing from diagnosis to epidemiologic surveillance has been indispensable in the past 5 years. The molecular testing with real‐time reverse transcription polymerase chain reaction (RT‐PCR) for the detection of SARS‐CoV‐2 was the reference standard for COVID‐19 diagnosis. Besides SARS‐CoV‐2 RT‐PCR testing, serological testing comprising the detection of IgM, IgA, or IgG antibodies to SARS‐CoV‐2‐specific epitopes has the potential to play an important role in the confirmation in individual patients with suspected COVID‐19 symptoms, or for the past SARS‐CoV‐2 infections [1]. Immune response to SARS‐CoV‐2 included cell‐mediated and antibody‐mediated immunity [2]. The spike (S) glycoproteins with their receptor‐binding domain (RBD) and the nucleocapsid (N) protein were widely used as the most common antigens in commercial serological assays for the detection of specific antibodies [3, 4]. In specific contexts, serological testing might be instrumental for acute diagnostic purposes, particularly when the RT‐PCR fails to identify SARS‐CoV‐2, for example, in patients who are greater than 14 days from their onset of symptoms [5]. Freund et al. reported that serological markers as part of medical follow‐up of symptomatic COVID‐19 patients can be used for prognostication; the study found anti‐S levels were significantly associated with previous severe COVID‐19 [6]. In addition, serological testing has been reported to be significant and important for personalized vaccination plans. Vaccines are designed to induce antibodies to the S antigen or RBD [7]; vaccine‐induced antibodies may arise in response to the S antigen and are, therefore, potentially detectable by any assay using the S antigen or RBD. Freund et al. also reported that the trajectory of anti‐S IgG levels after vaccination was found to predict the response to future COVID vaccinations, and the determination of the characteristics of the humoral response to COVID‐19 vaccinations is significant in predicting the humoral response to the booster vaccines [8]. Serological testing also has potential utility for tracking the course of the SARS‐CoV‐2 pandemic in the community. Screening of individuals who may be a source for prophylactic or therapeutic neutralizing antibodies is another application of serological testing [9]. Multiple manufacturers offered various high‐throughput serological assays differing not only in their antibody isotypes (i.e., IgA, IgM, IgG, or total antibody) but also targeted SARS‐CoV‐2 antigens (i.e., the S1 subunit of the spike protein, N protein, or RBD). Due to urgency and demand in the initial days of the COVID‐19 pandemic, numerous serological assays were rapidly developed and have been validated on a limited number of samples. The diagnostic efficacy of serological assays varies greatly; few studies were conducted to compare the performance of these assays on a large scale. This study aimed to evaluate the analytic performance and diagnostic characteristics of 11 commercial serological assays for the detection of SARS‐CoV‐2 specific IgG, IgM, and total antibodies. The 11‐assays comparison included the access SARS‐CoV‐2 IgG assay from Beckman Coulter (USA), EDI novel coronavirus COVID‐19 IgG and EDI novel coronavirus COVID‐19 IgM assays from Epitope Diagnostics (San Diego, CA, USA), iFlash‐SARS‐CoV‐2 IgG and iFlash‐SARS‐CoV‐2 IgM from Shenzhen YHLO Biotech (Shenzhen, China), MAGLUMI 2019‐nCoV IgG and MAGLUMI 2019‐nCoV IgM assays from Snibe Diagnostic (Shenzhen, China), ortho‐clinical anti‐SARS‐CoV‐2 IgG and ortho‐clinical anti‐SARS‐CoV‐2 total assays from Ortho Clinical Diagnostics (France), Siemens SARS‐CoV‐2 total (COV2T) assay from Siemens (Munich, Germany), and Wantai SARS‐CoV‐2 total Ab assay from Wantai Biological Pharmacy Enterprise (Beijing, China). Meanwhile we assessed the diagnostic accuracy of antibody isotypes by meta‐analysis and indirect comparison.

## 2 Materials and Methods

### 2.1 Search Strategy

Studies were identified by searching the Cochrane Library, PubMed, Embase, Web of Science, Chinese Biological Medicine Database (CBM), China National Knowledge Infrastructure (CNKI), WANFANG, and Chinese Weipu (VIP) databases. The search terms used were (“2019‐nCoV” OR “coronavirus disease 2019 virus” OR “2019 novel coronavirus” OR “COVID‐19” OR “COVID‐19 diagnostic testing” OR “COVID‐19 serological test” OR “SARS‐CoV‐2” OR “severe acute respiratory syndrome coronavirus 2” OR “Anti‐SARS‐CoV‐2”) AND (“Access SARS‐CoV‐2 IgG” OR “EDI Novel Coronavirus COVID‐19” OR “iFlash‐SARS‐CoV‐2” OR “MAGLUMI 2019‐nCoV” OR “Ortho‐Clinical anti‐SARS‐CoV‐2” OR “Siemens SARS‐CoV‐2” OR “Siemens SARS‐CoV‐2 Total (COV2T)” OR “Wantai SARS‐CoV‐2”). The searches were limited to articles published in Chinese or English.

### 2.2 Inclusion and Exclusion Criteria

We included studies that evaluated the performance of the above‐mentioned 11 anti‐SARS‐CoV‐2 antibody serological assays. The 11‐assay comparison for inclusion in the data analysis met the following inclusion criteria: (1) Studies which included the COVID‐19 patients' serum samples and negative control serum samples reporting both sensitivity and specificity of serological assays for COVID‐19; (2) the diagnosis of SARS‐CoV‐2 (COVID‐19) by taking RT‐PCR as the reference standard meanwhile based on clinical symptoms and imaging diagnosis; (3) stored pre‐COVID‐19 blood samples collected from the healthy blood donors and the individuals with a history of PCR‐confirmed non‐COVID‐19 infection within the previous 6 months were used as negative control; (4) the number of true positive (TP), true negative (TN), false positive (FP), and false negative (FN) were then abstracted, or data that could transform into above information were reported.

The exclusion criteria: (1) The studies evaluated the performance of in‐house developed antibody assay for the diagnosis of COVID‐19 instead of any commercial serological assay; (2) the studies evaluated the serological assays for the detection of antibodies generated by vaccines against SARS‐CoV‐2; (3) the studies whose COVID‐19 patients were diagnosed without at least one positive RT‐PCR test carried out; (4) studies in which serological assays were evaluated without providing enough information for the immunoglobulin classes (IgG, IgA, IgM, or total antibody), the targeting antigen, manufacturer/platform or the method; (5) studies with negative control sample sizes or patients serums samples less than 30.

### 2.3 Data Extraction and Quality Assessment

Articles were independently assessed for inclusion by the two authors of this paper (Ying Zhao and Minjie Zhang), and data from included studies were extracted using the Quality Assessment of Diagnostic Accuracy Studies (QUADAS‐2) tool for the domains of patient selection, performance of the index test, performance of the reference test, and flow and timing (for risk of bias only). The extracted data included the name of the first author of the article, publication year, manufacturer, method, assay, immunoglobulin isotypes (IgM, IgG, or total antibody), type of antigen (S, N, or RBD), COVID‐19 patients sample size, and the negative control sample size. The TP, FP, TN, and FN results of each arm were reported separately.

### 2.4 Statistical Analysis

The pooled sensitivity, specificity, positive likelihood ratio (PLR), negative likelihood ratio (NLR), diagnostic odds ratio (DOR), and the summary receiver operating characteristic curves (SROC) with corresponding 95% (confidence intervals, CI) were measured. The SROC curve (based on 2 × 2 contingency tables) was established to show the sensitivity and specificity for each individual arm, and the area under the curve (AUC) was used to determine diagnostic accuracy. Review Manager 5.3 (Cochrane Collaboration) analysis software was used to build the area under the SROC curve (AUC) graphics by making use of different colors for different serological assays. The relative diagnostic odds ratio (RDOR) of indirect comparison was used to compare the diagnostic accuracy of different assays and different immunoglobulin isotypes. The RDOR outcomes were summarized and exhibited in paired forest plots by R software (Parametric Technology Corporation). When the 95% CI of the RDOR contains 1, it indicates that the difference between the two comparison objects was not statistically significant; alternatively, when its 95% CI exceeds 1, suggesting that the difference between the two comparison objects was statistically significant. The Deek's test was used to evaluate whether there was publication bias.

## 3 Results

### 3.1 Study Characteristics

**Figure 1:**
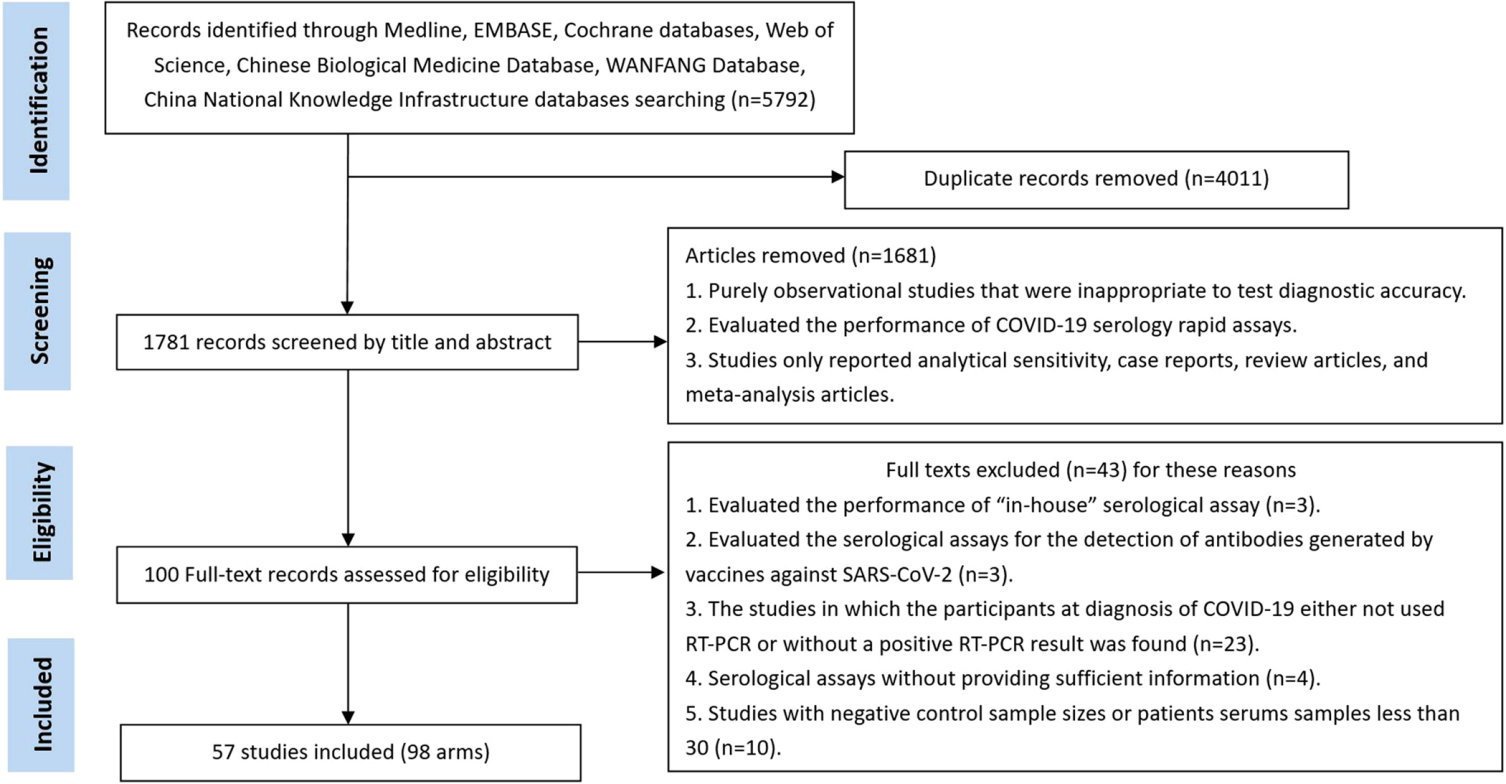
Flow diagram of selecting the literature and screening process.

A PRISMA flow chart in Figure 1 was used. A total of 1781 pieces of literature were identified after the removal of duplicate articles. One hundred after full‐text review were assessed for eligibility. Fifty‐seven articles were included finally in the systematic review [10, 11, 12, 13, 14, 15, 16, 17, 18, 19, 20, 21, 22, 23, 24, 25, 26, 27, 28, 29, 30, 31, 32, 33, 34, 35, 36, 37, 38, 39, 40, 41, 42, 43, 44, 45, 46, 47, 48, 49, 50, 51, 52, 53, 54, 55, 56, 57, 58, 59, 60, 61, 62, 63, 64, 65, 66]. The detailed characteristics of the articles included in this study are shown in Table 1.

**Table 1:** Characteristics of the included studies.

Author	Year	Method	Manufacturer/platform	Assay	Antigen	Antibody type	COVID‐19 patient samples (*n*)	Controls (*n*)
A Bown [10]	2020	CLIA	Siemens Atellica	Siemens SARS‐CoV‐2 total (COV2T)	S1	Total antibody	536	976
Al‐Jighefee HT [11]	2021	ELISA	NA	EDI novel coronavirus COVID‐19 IgG	N	IgG	291	119
		ELISA	NA	EDI novel coronavirus COVID‐19 IgM	N	IgM	291	119
Andrey DO [12]	2021	ELISA	DYNEX DSX	EDI novel coronavirus COVID‐19 IgG	N	IgG	172	185
Bundschuh C [13]	2020	ELISA	Serion Diagnostics	EDI novel coronavirus COVID‐19 IgG	N	IgG	104	456
		ELISA	Serion Diagnostics	EDI novel coronavirus COVID‐19 IgM	N	IgM	104	456
Chiereghin A [14]	2020	CLIA	iFlash 3000	iFlash‐SARS‐CoV‐2 IgG	N and S	IgG	207	130
		CLIA	iFlash 3000	iFlash‐SARS‐CoV‐2 IgM	N and S	IgM	207	130
Chua KYL [15]	2020	CLIA	Vitros 5600	Ortho‐clinical anti‐SARS‐CoV‐2 IgG	S1	IgG	86	95
		CLIA	Beckman Unicel DxI 800	Access SARS‐CoV‐2 IgG	S1‐RBD	IgG	86	95
Davidson N [16]	2020	ELISA	NA	EDI novel coronavirus COVID‐19 IgG	N	IgG	71	138
		ELISA	NA	EDI novel coronavirus COVID‐19 IgM	N	IgM	71	138
Deng Jielun [17]	2021	CLIA	iFlash 3000	iFlash‐SARS‐CoV‐2 IgG	N and S	IgG	69	73
		CLIA	iFlash 3000	iFlash‐SARS‐CoV‐2 IgM	N and S	IgM	69	73
Egger M [18]	2020	ELISA	NA	EDI novel coronavirus COVID‐19 IgG	N	IgG	104	200
		ELISA	NA	EDI novel coronavirus COVID‐19 IgM	N	IgM	104	200
Florin L [19]	2021	CLIA	Siemens Atellica IM1300	Siemens SARS‐CoV‐2 total (COV2T)	S1‐RBD	Total Ab	175	90
Garnett E [20]	2020	CLIA	Vitros 5600	Ortho‐clinical anti‐SARS‐CoV‐2 total	S	Total antibody	79	57
Gdoura M [21]	2022	CLIA	Beckman Coulter	Access SARS‐CoV‐2 IgG	S1‐RBD	IgG	72	119
Han Xiaoyan [22]	2023	CLIA	iFlash 3000	iFlash‐SARS‐CoV‐2 IgG	N and S	IgG	123	41
		CLIA	iFlash 3000	iFlash‐SARS‐CoV‐2 IgM	N and S	IgM	123	41
Harritshøj LH [23]	2021	CLIA	Siemens Atellica IM	Siemens SARS‐CoV‐2 total (COV2T)	S1	Total antibody	148	596
		CLIA	Siemens Dimension Vista 500	Siemens SARS‐CoV‐2 total (COV2T)	S1	Total antibody	147	596
		CLIA	Maglumi 4000+	MAGLUMI 2019‐nCoV IgG	Unspecified	IgG	148	1173
		CLIA	Maglumi 800	MAGLUMI 2019‐nCoV IgM	Unspecified	IgM	150	1184
		CLIA	Vitros 3600	Ortho‐clinical anti‐SARS‐CoV‐2 IgG	S1	IgG	150	600
		CLIA	Vitros 3600	Ortho‐clinical anti‐SARS‐CoV‐2 total	S1	Total antibody	150	605
		ELISA	Tecan Sunrise	Wantai SARS‐CoV‐2 total Ab	S	Total antibody	150	659
		CLIA	iFlash 1800	iFlash‐SARS‐CoV‐2 IgG	N and S	IgG	150	586
		CLIA	iFlash 1800	iFlash‐SARS‐CoV‐2 IgM	N and S	IgM	150	585
Heffernan E [24]	2021	ELISA	Dynex DS2	Wantai SARS‐CoV‐2 total Ab	S	Total antibody	137	100
Herroelen PH [25]	2020	ELISA	Bio‐Rad Version EIA 0_16	Wantai SARS‐CoV‐2 total Ab	S1‐RBD	Total antibody	169	57
Hörber S [26]^a^	2020	CLIA	Siemens ADVIA Centaur XPT	Siemens SARS‐CoV‐2 total (COV2T)	S1	Total antibody	186	123
Horn MP [27]	2022	ELISA	DYNEX DSX	EDI novel coronavirus COVID‐19 IgG	N	IgG	192	3462
Igawa G [28]	2021	CLIA	Siemens Dimension EXL 200	Siemens SARS‐CoV‐2 total (COV2T)	S1‐RBD	Total Ab	236	98
Ikegami S [29]	2021	CLIA	Beckman Coulter	Access SARS‐CoV‐2 IgG	S1‐RBD	IgG	97	100
Irsara C [30]	2021	CLIA	Siemens ADVIA Centaur XPT	Siemens SARS‐CoV‐2 total (COV2T)	S1	Total antibody	195	288
Kubota K [31]	2021	CLIA	Vitros 3600	Ortho‐clinical anti‐SARS‐CoV‐2 IgG	S	IgG	66	148
		CLIA	Vitros 3600	Ortho‐clinical anti‐SARS‐CoV‐2 total	S1	Total antibody	66	148
Kundu D [32]	2022	CLIA	Siemens ADVIA Centaur XPT	Siemens SARS‐CoV‐2 total (COV2T)	S1	Total antibody	153	150
Lapić I [33]	2020	CLIA	Maglumi 800	MAGLUMI 2019‐nCoV IgG	N and S	IgG	42	48
Lester SN [34]	2024	CLIA	Vitros ECi/ECiQ/3600 and Vitros 5600/XT 7600	Ortho‐clinical anti‐SARS‐CoV‐2 total	S1	Total antibody	87	117
Li Ping [35]	2020	CLIA	iFlash 3000	iFlash‐SARS‐CoV‐2 IgG	N and S	IgG	116	134
		CLIA	iFlash 3000	iFlash‐SARS‐CoV‐2 IgM	N and S	IgM	116	134
Mafi S [36]	2023	ELISA	NA	Wantai SARS‐CoV‐2 total Ab	S1	Total antibody	110	120
Mairesse A [37]	2020	CLIA	iFlash 1800	iFlash‐SARS‐CoV‐2 IgG	N and S	IgG	178	75
		CLIA	iFlash 1800	iFlash‐SARS‐CoV‐2 IgM	N and S	IgM	178	75
Manthei DM [38]	2021	CLIA	Siemens Centaur XP	Siemens SARS‐CoV‐2 total (COV2T)	S1‐RBD	Total Ab	131	188
Marlet J [39]	2020	ELISA	NA	Wantai SARS‐CoV‐2 total Ab	S	Total Ab	58	89
Montesinos I [40]	2020	CLIA	Maglumi 800	MAGLUMI 2019‐nCoV IgG	Unspecified	IgG	126	72
		CLIA	Maglumi 800	MAGLUMI 2019‐nCoV IgM	Unspecified	IgM	126	72
Naaber P [41]	2020	CLIA	Maglumi 1000	MAGLUMI 2019‐nCoV IgG	Unspecified	IgG	97	100
		ELISA	Dynex Agility	EDI novel coronavirus COVID‐19 IgG	N	IgG	97	100
Nedelcu I [42]	2021	ELISA	Dynex DSX	EDI novel coronavirus COVID‐19 IgG	N	IgG	528	161
		ELISA	Dynex DSX	EDI novel coronavirus COVID‐19 IgM	N	IgM	528	161
Nicholson S [43]	2021	ELISA	NA	Wantai SARS‐CoV‐2 total Ab	S1	Total antibody	96	209
Nyagwange J [44]	2022	ELISA	NA	Wantai SARS‐CoV‐2 total Ab	S1	Total antibody	149	327
Oved K [45]	2020	CLIA	Beckman Coulter	Access SARS‐CoV‐2 IgG	S1‐RBD	IgG	162	318
	2020	CLIA	Siemens ADVIA Centaur XPT	Siemens SARS‐CoV‐2 total (COV2T)	S1‐RBD	Total Ab	156	432
Padoan A [46]	2020	CLIA	Vitros ECi/ECiQ/3600 and Vitros 5600/XT 7600	Ortho‐clinical anti‐SARS‐CoV‐2 IgG	S	IgG	130	54
		CLIA	Vitros ECi/ECiQ/3600 and Vitros 5600/XT 7600	Ortho‐clinical anti‐SARS‐CoV‐2 total	S1	Total antibody	130	54
Parai D [47]	2021	CLIA	iFlash 1800	iFlash‐SARS‐CoV‐2 IgG	N and S	IgG	594	100
Pérez‐García F [48]	2021	CLIA	Siemens Atellica	Siemens SARS‐CoV‐2 total (COV2T)	S1	Total antibody	50	60
Pflüger LS [49]	2020	CLIA	Siemens Atellica	Siemens SARS‐CoV‐2 total (COV2T)	S1	Total Ab	75	320
		ELISA	Euroimmun Analyzer I‐2 P	Wantai SARS‐CoV‐2 total Ab	S1	Total Ab	75	320
Piec I [50]	2021	ELISA	Dynex Agility	EDI novel coronavirus COVID‐19 IgG	N	IgG	43	152
Riester E [51]	2021	CLIA	iFlash 1800	iFlash‐SARS‐CoV‐2 IgG	N and S	IgG	104	928
		CLIA	iFlash 1800	iFlash‐SARS‐CoV‐2 IgM	N and S	IgM	104	928
Rikhtegaran Tehrani Z [52]	2020	ELISA	NA	EDI novel coronavirus COVID‐19 IgG	N	IgG	97	288
		ELISA	NA	EDI novel coronavirus COVID‐19 IgM	N	IgM	95	299
Sekirov I [53]	2021	CLIA	Vitros XT 7600	Ortho‐clinical anti‐SARS‐CoV‐2 IgG	S	IgG	42	65
		CLIA	Vitros XT 7600	Ortho‐clinical anti‐SARS‐CoV‐2 total	S1	Total antibody	42	65
		CLIA	Siemens ADVIA Centaur XPT	Siemens SARS‐CoV‐2 total (COV2T)	S1	Total antibody	42	65
Şener B [54]	2022	CMIA	Beckman Coulter	Access SARS‐CoV‐2 IgG	S1‐RBD	IgG	125	50
		CMIA	Siemens Atellica	Siemens SARS‐CoV‐2 total (COV2T)	S1‐RBD	Total Ab	131	50
Serre‐Miranda C [55]	2021	CLIA	NA	MAGLUMI 2019‐nCoV IgG	N and S	IgG	117	35
		CLIA	NA	MAGLUMI 2019‐nCoV IgM	N and S	IgM	117	35
Soleimani R [56]	2021	CLIA	Maglumi 800	MAGLUMI 2019‐nCoV IgG	N and S	IgG	176	100
	2021	CLIA	Maglumi 800	MAGLUMI 2019‐nCoV IgM	N and S	IgM	176	100
Syre H [57]	2022	ELISA	DYNEX DS2 system	Wantai SARS‐CoV‐2 total Ab	S	Total antibody	211	320
Tan SS [58]	2020	CLIA	Beckman Unicel DxI 800	Access SARS‐CoV‐2 IgG	S1‐RBD	IgG	173	163
		CLIA	Siemens ADVIA Centaur XPT	Siemens SARS‐CoV‐2 total (COV2T)	S1	Total antibody	173	163
		CLIA	Vitros 3600	Ortho‐clinical anti‐SARS‐CoV‐2 total	S1	Total antibody	173	163
Theel ES [59]	2020	ELISA	Dynex Agility	EDI novel coronavirus COVID‐19 IgG	N	IgG	61	149
		CLIA	Vitros 3600	Ortho‐clinical anti‐SARS‐CoV‐2 IgG	S	IgG	61	149
Tolan NV [60]	2023	ELISA	NA	EDI novel coronavirus COVID‐19 IgG	N	IgG	105	55
		ELISA	NA	EDI novel coronavirus COVID‐19 IgM	N	IgM	103	57
Van Elslande J [61]	2020	CLIA	Maglumi 800	MAGLUMI 2019‐nCoV IgG	Unspecified	IgG	223	113
Velay A [62]	2020	ELISA	NA	EDI novel coronavirus COVID‐19 IgG	N	IgG	198	100
		ELISA	NA	EDI novel coronavirus COVID‐19 IgM	N	IgM	198	100
Ward MD [63]	2021	CLIA	Siemens Atellica	Siemens SARS‐CoV‐2 total (COV2T)	S1	Total antibody	112	2030
		CLIA	Siemens EXL	Siemens SARS‐CoV‐2 total (COV2T)	S1	Total antibody	112	2030
Wu Lianpeng [64]	2021	CLIA	iFlash 3000	iFlash‐SARS‐CoV‐2 IgG	N and S	IgG	179	50
		CLIA	iFlash 3000	iFlash‐SARS‐CoV‐2 IgM	N and S	IgM	179	50
Yassine HM [65]	2021	ELISA	Epoch 2	EDI novel coronavirus COVID‐19 IgG	N	IgG	101	70
Zilla M [66]	2021	CLIA	Beckman Coulter	Access SARS‐CoV‐2 IgG	S1‐RBD	IgG	154	184
		CLIA	Siemens Centaur XP	Siemens SARS‐CoV‐2 total (COV2T)	S1	Total antibody	154	184
		CLIA	Siemens Vista 1500	Siemens SARS‐CoV‐2 total (COV2T)	S1	Total antibody	154	184

^a^ If borderline results were considered positive.

Abbreviations: CLIA, chemiluminescent immunoassay; ELISA, enzyme immunoassay; N, nucleocapsid antigen; NA, not specified; RBD, receptor‐binding domain; S1, S1 domain of viral spike protein; S1/S2, recombinant S1 and S2 antigens.

### 3.2 Quality Assessment

**Figure 2:**
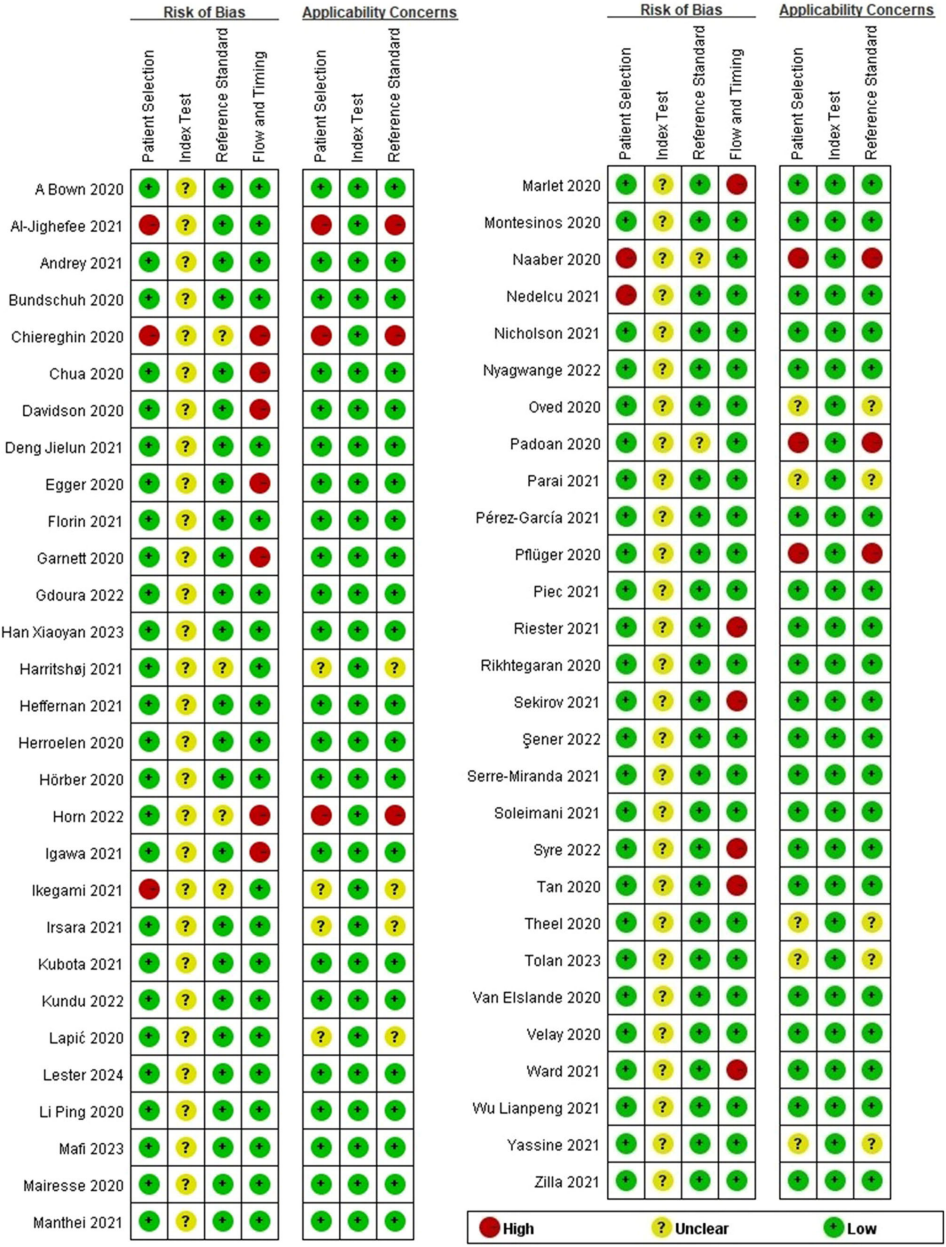
Risk of bias and applicability concerns summary.

The assessment was performed using the Review Manager Software version 5.3. Figure 2 summarized the QUADA‐2 assessment. Five articles were judged as “high” in the patient selection domain of the risk of bias (including Al‐Jighefee, Chiereghin, Ikegami, Naaber, and Nedelcu). Among the above five articles, two or three questions of a domain were answered as “no”; therefore, the level of risk of bias was judged as “high” in the patient selection domain. For the index test domain of the risk of bias, it was found that the first question of the domain was answered as “no” on account of the serological assays being evaluated not in blind; therefore, all articles were judged as “unclear”. Six articles (including Chiereghin, Harritshøj, Horn, Ikegami, Naaber, and Padoan) whose patients had been diagnosed with COVID‐19 by positive SARS‐CoV‐2 RT‐PCR regardless of clinical symptoms or contained recovered COVID‐19 patients were judged as “unclear” in the reference standard domain of the risk of bias. Thirteen articles (including Chiereghin, Chua, Davidson, Egger, Garnett, Horn, Igawa, Marlet, Riester, Sekirov, Syre, Tan, and Ward) were judged as “high” in the flow and timing domain. The applicability judgment results for the patient selection domain were consistent with the reference standard domain; six articles (including Al‐Jighefee, Chiereghin, Horn, Naaber, Padoan, and Pflüger), including asymptomatic COVID‐19 patients were judged as “high,” and nine articles (including Harritshøj, Ikegami, Irsara, Lapić, Oved, Parai, Theel, Tolan, and Yassine) including recovered COVID‐19 patients were judged as “unclear.”

### 3.3 Data Synthesis and Meta‐Analysis

**Figure 3:**
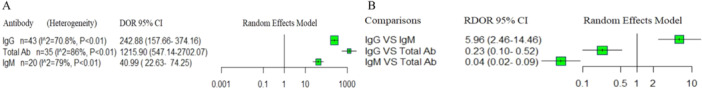
(A) Pooled DOR with 95% CIs of immunoglobulin classes. (B) Indirect comparison forest plots of RDOR with 95% CIs for all three pairwise immunoglobulin comparisons. CIs, confidence intervals; DOR, diagnostic odds ratio; RDOR, relative diagnostic odds ratio.

First, we evaluated the diagnostic accuracy of immunoglobulin isotypes (IgM, IgG, and total antibody). Due to significant heterogeneity in the analysis of three immunoglobulin isotypes, a random effects model was used. A forest plot of DOR with 95% CIs for immunoglobulin isotypes is shown in Figure 3A. The pooled DOR for IgG (242.88 [95% CI 157.66−374.16]), IgM (40.99 [95% CI 22.63−74.25]), and total antibody (1215.90 [95% CI 547.14−2702.07]) showed that assays‐based total antibody had the better diagnostic accuracy compared to assays‐based IgG and assays‐based IgM with significant difference. Moreover, indirect comparison results of RDOR with 95% CIs showed that assays‐based total antibody and assays‐based IgG showed significantly better diagnostic accuracy than assays‐based IgM (as shown in Figure 3B).

**Figure 4:**
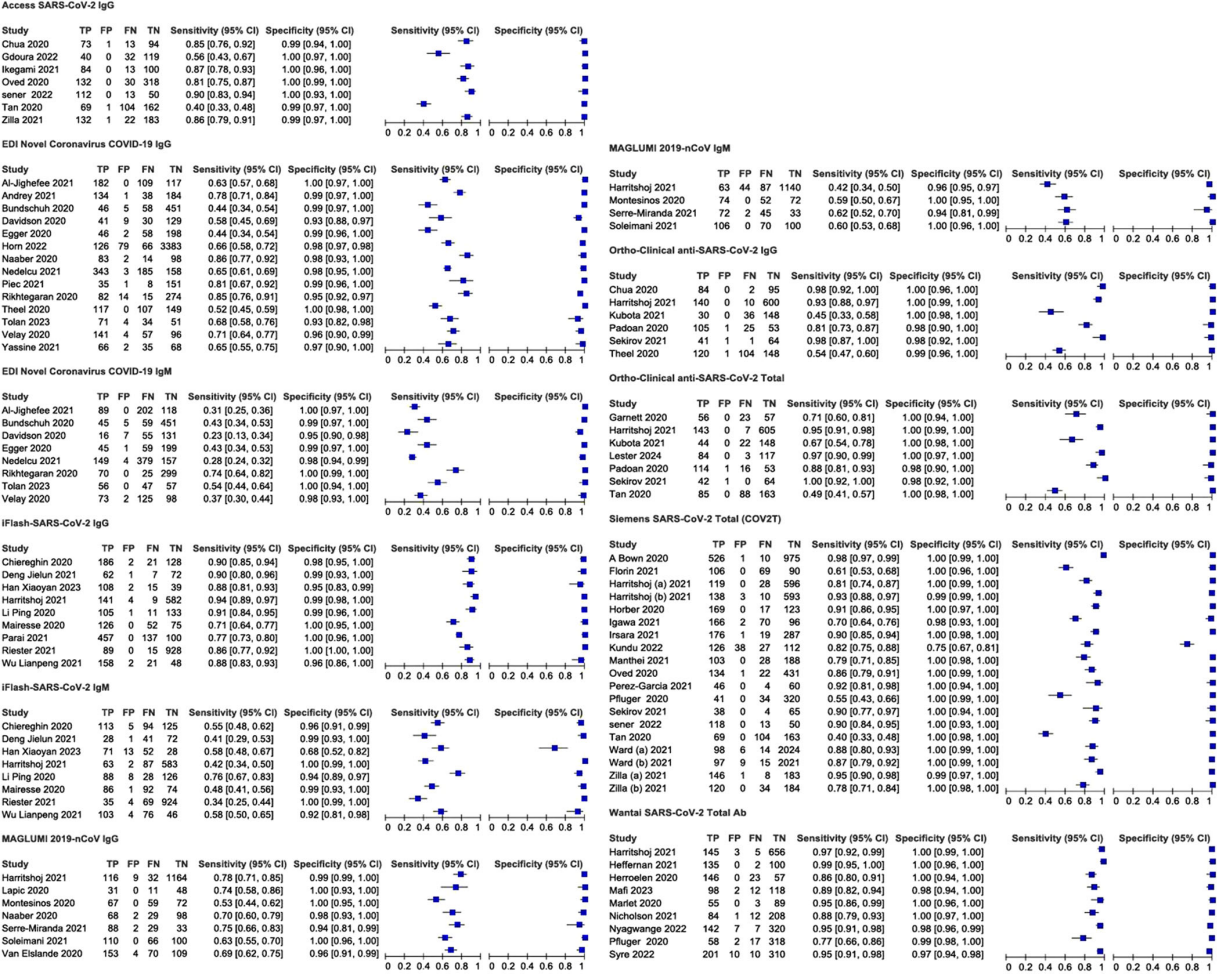
Forest plots of coupled sensitivity and specificity with 95% confidence intervals for 11 serological assays. Harritshøj (A): Siemens Vista assay was performed, Harritshøj (B): Siemens Atellica assay was performed; Ward (A): Siemens Atellica assay was used, Ward (B): Siemens EXL systems were used; Zilla (A): Siemens Centaur assay was performed; Zilla (B): Siemens Vista assay was performed.

**Figure 5:**
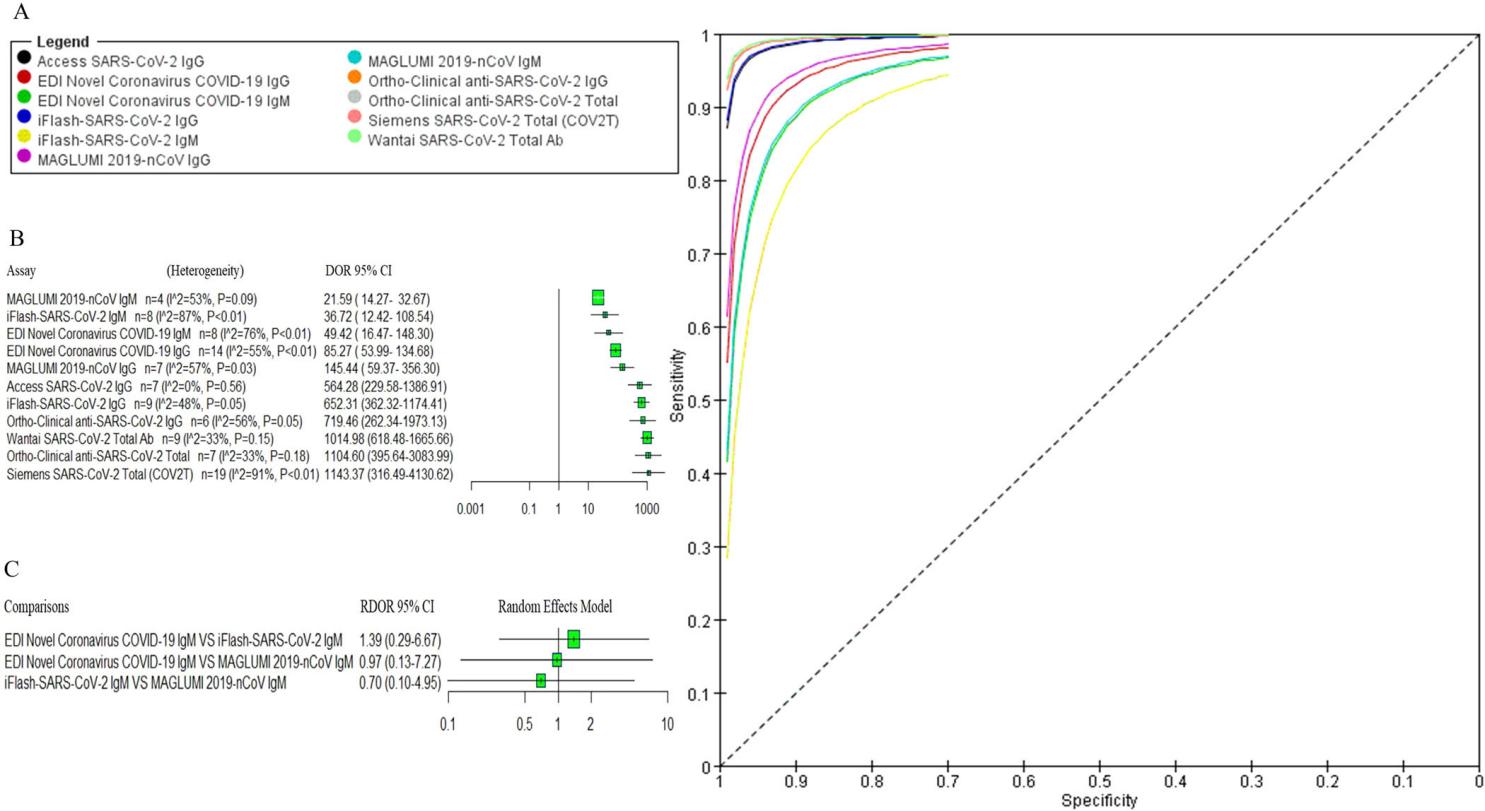
(A) SROC curve of the 11 serological assays. (B) Pooled DOR with 95% CIs of the 11 serological assays. (C) Indirect comparison forest plots of RDOR with 95% CIs for three IgM assays pairwise comparisons. CIs, confidence intervals; DOR, diagnostic odds ratio; RDOR, relative diagnostic odds ratio; SROC, summary receiver operating characteristics.

**Figure 6:**
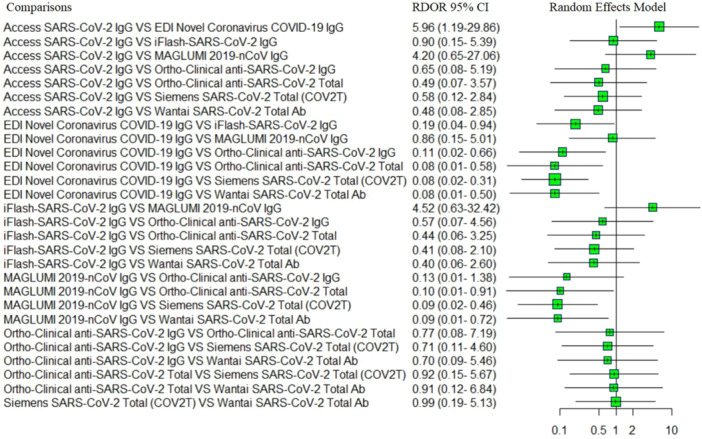
Indirect comparison forest plots of RDOR with 95% CIs for IgG and total antibody assays pairwise comparisons. CIs, confidence intervals; RDOR, relative diagnostic odds ratio.

As mentioned above, the diagnostic efficacy of the assays‐based total antibody had statistically significantly higher accuracy than those of assays‐based IgG and assays‐based IgM. We assessed the diagnostic accuracy of assays‐based IgM separately. Meta‐analyses evaluating the parameters of the accuracy of the reported assays were performed, and results are shown in Table 2. Forest plots of coupled sensitivity and specificity with 95% CIs for 11 serological assays are shown in Figure 4. We also constructed the SROC curves for all 11 serological assays (as shown in Figure 5A). The pooled DOR results of 11 serological assays were shown by forest plots (as shown in Figure 5B). There was no significant heterogeneity for access SARS‐CoV‐2 IgG, iFlash‐SARS‐CoV‐2 IgG, MAGLUMI 2019‐nCoV IgM, ortho‐clinical anti‐SARS‐CoV‐2 IgG, ortho‐clinical anti‐SARS‐CoV‐2 total, and Wantai SARS‐CoV‐2 total Ab, and a fixed effects model was used. The results of RDOR with 95% CIs were exhibited in the indirect comparison forest plot by R software. From the pooled DOR and the SROC curves, the overall diagnostic accuracy of the Siemens SARS‐CoV‐2 total (COV2T) (1143.37 [95% CI 316.49−4130.62]), ortho‐clinical anti‐SARS‐CoV‐2 total (1104.60 [95% CI 395.64−3083.99]), Wantai SARS‐CoV‐2 total Ab (1014.98 [95% CI 618.48−1665.66]), and ortho‐clinical anti‐SARS‐CoV‐2 IgG (719.46 [95% CI 262.34−1973.13]) performed better than the other serological assays; indirect comparison results of RDOR with 95% CIs for these four pairwise assays showed that there was no significant difference between them. Meanwhile, the RDOR results suggested that the diagnostic accuracy of these four assays was statistically significantly higher than EDI novel coronavirus COVID‐19 IgG. The RDOR value of EDI novel coronavirus COVID‐19 IgG versus iFlash‐SARS‐CoV‐2 IgG was 0.19 (95% CI 0.04−0.94), which suggested that the diagnostic accuracy of iFlash‐SARS‐CoV‐2 IgG was statistically significantly higher than EDI novel coronavirus COVID‐19 IgG (as shown in Figure 6). The diagnostic accuracy of the three IgM assays had no significant difference (as shown in Figure 5C).

**Table 2:** Summary table of the diagnostic accuracy.

Assay	Pooled analysis results (95% CI)						Heterogeneity		Deek's test	
	Pooled sensitivity	Pooled specificity	Pooled PLR	Pooled NLR	AUC	DOR	*I* ^2^ (%)	*p*	*t*	*p*
Access SARS‐CoV‐2 IgG	0.78 (0.64−0.87)	1.00 (0.99−1.00)	275.20 (82.90−914.00)	0.22 (0.13−0.38)	1.00	564.28 (229.58−1386.91)^a^	0	0.56	−0.53	0.618
EDI novel coronavirus COVID‐19 IgG	0.67 (0.60−0.74)	0.98 (0.97−0.99)	36.10 (21.90−59.50)	0.34 (0.27−0.41)	0.95	85.27 (53.99−134.68)	55	< 0.01	−0.18	0.859
EDI novel coronavirus COVID‐19 IgG (omitting Davidson)	0.68 (0.60−0.74)	0.98 (0.97−0.99)	38.20 (24.10−60.70)	0.33 (0.26−0.41)	0.97	87.61 (67.43−113.81)^a^	32	0.13	0.25	0.806
EDI novel coronavirus COVID‐19 IgM	0.41 (0.30−0.52)	0.99 (0.97−1.00)	47.20 (12.80−173.40)	0.60 (0.49−0.73)	0.86	49.42 (16.47−148.30)	76	< 0.01	0.32	0.758
EDI novel coronavirus COVID‐19 IgM (omitting Davidson)	0.44 (0.33−0.55)	0.99 (0.98−1.00)	63.60 (18.40−219.30)	0.57 (0.46−0.70)	0.91	67.86 (25.55−180.22)	57	0.03	1.12	0.315
iFlash‐SARS‐CoV‐2 IgG	0.87 (0.82−0.90)	1.00 (0.98−1.00)	195.10 (44.00−865.00)	0.13 (0.10−0.18)	0.97	652.31 (362.32−1174.41)^a^	48	0.05	−2.52	0.040^b^
iFlash‐SARS‐CoV‐2 IgM	0.52 (0.49−0.55)	0.98 (0.97−0.99)	18.37 (5.71−59.05)	0.52 (0.45−0.60)	0.76	36.72 (12.42−108.54)	87	< 0.01	−3.41	0.014^b^
iFlash‐SARS‐CoV‐2 IgM (omitting Han Xiaoyan)	0.50 (0.41−0.60)	0.98 (0.96−0.99)	32.30 (15.70−66.20)	0.50 (0.42−0.61)	0.86	49.19 (31.92−75.81)^a^	51	0.06	−2.37	0.064
MAGLUMI 2019‐nCoV IgG	0.69 (0.62−0.74)	0.99 (0.97−1.00)	71.30 (22.80−223.00)	0.32 (0.26−0.38)	0.90	145.44 (59.37−356.30)	57	0.03	−1.26	0.263
MAGLUMI 2019‐nCoV IgG (omitting Harritshøj)	0.66 (0.60−0.72)	0.99 (0.95−1.00)	59.30 (13.40−261.50)	0.34 (0.29−0.40)	0.83	83.80 (42.86−163.85)^a^	0	0.72	0.12	0.912
MAGLUMI 2019‐nCoV IgM	0.55 (0.51−0.59)	0.97 (0.96−0.98)	23.77 (4.96−113.88)	0.45 (0.36−0.57)	0.59	21.59 (14.27−32.67)^a^	53	0.09	0.74	0.534
Ortho‐clinical anti‐SARS‐CoV‐2 IgG	0.86 (0.64−0.96)	1.00 (0.99−1.00)	330.20 (69.80−1562.30)	0.14 (0.04−0.42)	1.00	719.46 (262.34−1973.13)^a^	56	0.05	−0.68	0.536
Ortho‐clinical anti‐SARS‐CoV‐2 IgG (omitting Harritshøj)	0.85 (0.54‐0.96)	0.99 (0.98‐1.00)	139.20 (44.90−431.50)	0.16 (0.04−0.56)	1.00	459.87 (156.30−1353.00)^a^	33	0.20	1.13	0.340
Ortho‐clinical anti‐SARS‐CoV‐2 total	0.88 (0.70−0.96)	1.00 (0.98−1.00)	1036.40 (55.80−19258.80)	0.12 (0.04−0.34)	1.00	1104.60 (395.64−3083.99)^a^	33	0.18	−1.28	0.256
Siemens SARS‐CoV‐2 total (COV2T)	0.85 (0.78−0.90)	1.00 (0.99−1.00)	521.80 (135.30−2011.80)	0.15 (0.10−0.22)	0.99	1143.37 (316.49−4130.62)	91	< 0.01	−4.11	0.001^b^
Siemens SARS‐CoV‐2 total (COV2T) (omitting Kundu)	0.85 (0.78−0.90)	1.00 (1.00−1.00)	301.60 (196.80−462.10)	0.15 (0.10−0.22)	1.00	1581.39 (837.31−2986.72)	45	0.02	−4.90	0.000^b^
Wantai SARS‐CoV‐2 total Ab	0.93 (0.88−0.96)	0.99 (0.98−1.00)	107.80 (50.80−229.00)	0.07 (0.04−0.12)	1.00	1014.98 (618.48−1665.66)^a^	33	0.15	−0.10	0.919

^a^ The pooled DOR was calculated with a fixed effects model.

^b^ Significant publication bias was observed.

### 3.4 Heterogeneity Test and Sensitivity Analysis

We investigated heterogeneity for 11 serological assays; significant high heterogeneity was observed for EDI novel coronavirus COVID‐19 IgG, EDI novel coronavirus COVID‐19 IgM, iFlash‐SARS‐CoV‐2 IgM, MAGLUMI 2019‐nCoV IgG, and Siemens SARS‐CoV‐2 total. To determine the possible source of heterogeneity, sensitivity analysis was performed. Omitting a single study did not significantly affect the pooled DOR. Nevertheless, no significant heterogeneity was observed for EDI novel coronavirus COVID‐19 IgG (*p* = 0.13, *I* ^2^ = 32%) when the study “Davidson 2020” was removed as well as for iFlash‐SARS‐CoV‐2 IgM (*p* = 0.06, *I* ^2^ = 51%) when study “Han Xiaoyan 2023” was removed and for ortho‐clinical anti‐SARS‐CoV‐2 IgG (*p* = 0.20, *I* ^2^ = 33%) when study “Harritshøj 20213” was removed. The heterogeneity decreased for EDI novel coronavirus COVID‐19 IgM (*p* = 0.03, *I* ^2^ = 57%) when study “Davidson 2020” was removed as well as for Siemens SARS‐CoV‐2 total (*p* = 0.02, *I* ^2^ = 45%) when study “Kundu 2022” was removed. While “Harritshøj 2021” was the primary cause of the heterogeneity for MAGLUMI 2019‐nCoV IgG (*p* = 0.72, *I* ^2^ = 0%, omitting “Harritshøj 2021”) (as shown in Table 2).

### 3.5 Risk of Bias Assessment

The Deek's test was performed to detect publication bias for iFlash‐SARS‐CoV‐2 IgG, iFlash‐SARS‐CoV‐2 IgM, and Siemens SARS‐CoV‐2 total, and the results of publication bias showed that the *p* value for aromatase was < 0.05, indicating that significant publication bias was observed (as shown in Table 2).

## 4 Discussion

Since the start of the COVID‐19 pandemic, an increasing number of serological SARS‐CoV‐2 assays have been introduced to the diagnostic market. We have demonstrated a comprehensive evaluation of 11 commercially available anti‐SARS‐CoV‐2 antibody assays. First, we evaluated the diagnostic efficiency of eight assays for detecting IgG and total antibodies against SARS‐CoV‐2. Taking into account the manufacturer's threshold, the pooled sensitivity among the evaluated eight assays ranged from 67% to 93%. Wantai SARS‐CoV‐2 total Ab and ortho‐clinical anti‐SARS‐CoV‐2 total assays had the best pooled sensitivity, followed by the iFlash‐SARS‐CoV‐2 IgG, ortho‐clinical anti‐SARS‐CoV‐2 IgG, and Siemens SARS‐CoV‐2 total (COV2T) assays. The high sensitivity of the two assays could be due to the ability of the two assays to detect all immunoglobulin classes. The pooled sensitivity of the EDI Novel Coronavirus COVID‐19 IgG and MAGLUMI 2019‐nCoV IgG assays was the lowest with a positive rate of 67% and 69% in the neglect of the three assays‐based IgM conditions, the pooled sensitivity of the three IgM assays varied from 41% to 55%. The overall sensitivity of the IgM assays was low, suggesting that there was limited utility in testing for IgM assays. Other studies have suggested development of IgM may occur earlier using a nucleocapsid antigen target compared to the spike glycoproteins [67, 68]. The pooled specificity of MAGLUMI 2019‐nCoV IgM was low compared to the other assays; all other assays demonstrated pooled specificity exceeding 98%. Besides the sensitivity and specificity, the pooled PLR (1036.40 and 521.80) and pooled NLR (0.12 and 0.15) for ortho‐clinical anti‐SARS‐CoV‐2 total and Siemens SARS‐CoV‐2 Total (COV2T) assays were also better than those for the other assays. Nevertheless, the pooled NLR (0.07) for Wantai SARS‐CoV‐2 total Ab displayed the best performance. The pooled DOR of Siemens SARS‐CoV‐2 total (COV2T), ortho‐clinical anti‐SARS‐CoV‐2 total, and Wantai SARS‐CoV‐2 total Ab assays were sharply higher compared to the other assays. We also constructed the SROC curves using RevMan 5.3 software and calculated the AUC using STATA software (version 12). The AUC was 1.00 for access SARS‐CoV‐2 IgG, ortho‐clinical anti‐SARS‐CoV‐2 IgG, ortho‐clinical anti‐SARS‐CoV‐2 total, and Wantai SARS‐CoV‐2 total Ab, 0.99 for Siemens SARS‐CoV‐2 total (COV2T). The results of the SROC curve and the AUC suggested that the diagnostic accuracy of those three assays (Siemens SARS‐CoV‐2 total, ortho‐clinical anti‐SARS‐CoV‐2 total, and Wantai SARS‐CoV‐2 total Ab) were relatively higher than the other assays.

Subsequently, we used RT‐PCR as the reference standard and conducted an indirect comparison between the 11 assays by calculating the RDOR value using R software. The adjustment indirect comparison forest plots of RDOR showed that the diagnostic accuracy of the four assays (Siemens SARS‐CoV‐2 total, ortho‐clinical anti‐SARS‐CoV‐2 total, Wantai SARS‐CoV‐2 total Ab, and ortho‐clinical anti‐SARS‐CoV‐2 IgG) had no significant difference and we also did not observe a significant difference between the other three assays (EDI novel coronavirus COVID‐19 IgM, iFlash‐SARS‐CoV‐2 IgM, and MAGLUMI 2019‐nCoV IgM) in the diagnostic accuracy of COVID‐19. We also constructed an indirect comparison to compare the diagnostic accuracy of immunoglobulin classes recognized (IgM, IgG, and total antibody). In our study, the pooled DOR of assays‐based IgM were low compared to the assays‐based IgG and the assays‐based total antibody. Independent of the serological method, the diagnostic performance of the IgM‐specific assays was lower than that of IgG and total antibody‐specific assays. Another study reported that SARS‐CoV‐2‐specific IgM is detected mostly in the early infection phase but only in rare cases [69, 70]. The antibodies assessed in these assays refer to structural antigenic proteins of SARS‐CoV‐2; the 11 serological assays differ in the type of immunoglobulin classes recognized as well as the nature of the antigen used for antibody recognition. At present, many studies have performed a structured systematic review and meta‐analysis to evaluate the diagnostic characteristics of serological testing for the detection of SARS‐CoV‐2 antibodies. Most of them provided the pooled analysis results (e.g., sensitivities and specificities) regarding the accuracy parameters of the reported serological assays. There are a limited number of comparable serological assays in the studies performed head‐to‐head comparisons. Under the condition of insufficient direct comparative study, we conducted an indirect comparison of the diagnostic efficacy of 11 assays; our data provide the overall diagnostic efficacy of 11 assays, as well as the antibody isotypes. Additionally, it is important to point out that our study took RT‐PCR as a reference standard and conducted an indirect comparison to compare the efficacy of antibody assays by calculating the RDOR value between them. To visualize results, we provide forest plots showing the RDOR with 95% CI of the 11‐assay comparison by R software.

This meta‐analysis also had some limitations. Due to the included studies differing in terms of method, manufacturer, and period of blood collection, we found high heterogeneity rates among trials. The expression change of antibodies against SARS‐CoV‐2 and the methods used for the detection of antibodies may have an effect on the overall diagnostic accuracy of serological assays. Because few articles included in this meta‐analysis have provided the data regarding the TP, FP, FN, and TN values at different sampling times, we could not directly address whether or not the sampling time affects the assay performance. The second is related to the methodological qualities of the primary studies. In the eligibility criteria, we did not set very strict definitions for the diagnosis of COVID‐19; in some studies, asymptomatic COVID‐19 and recovered COVID‐19 patient's serum samples comprise a large proportion. Specifically, the methods used for diagnosing COVID‐19 were not described in most studies. Another limitation was that this meta‐analysis had high heterogeneity, and sensitivity analysis indicated that the heterogeneity may be derived from a single study.

## 5 Conclusions

This study suggested that the Siemens SARS‐CoV‐2 total (COV2T), ortho‐clinical anti‐SARS‐CoV‐2 total, and Wantai SARS‐CoV‐2 total had high diagnostic efficiency. The diagnostic efficacy of the assays‐based total antibody had statistically significantly higher accuracy than those of assays‐based IgG and assays‐based IgM for COVID‐19.

## Author Contributions

**Ying Zhao** and **Minjie Zhang:** conception, design, and administrative support. **Weiwei Liang, Lijiang Fang,** and **Ying Zhao:** data analysis and interpretation. **Minjie Zhang, Weiwei Liang,** and **Lijiang Fang:** manuscript writing, collection, and assembly of data. All authors read and approved the final manuscript.

## Conflicts of Interest

The authors declare no conflicts of interest.

## Data Availability

Requests for any data can be made to the authors.
